# Facemask and Rapid Maxillary Expansion With Alternative Rapid Maxillary Expansion and Constriction Protocol in the Management of Skeletal Class III Malocclusion

**DOI:** 10.7759/cureus.50764

**Published:** 2023-12-19

**Authors:** Mohanakrishnan P J, Vijayadhith Chinnapan, Ashok Pothuri, Kaviya S, Catherine S Frank

**Affiliations:** 1 Orthodontics and Dentofacial Orthopaedics, Priyadarshini Dental College and Hospital, Tiruvallur, IND; 2 Orthodontics and Dentofacial Orthopaedics, Vivekanandha Dental College for Women, Chennai, IND

**Keywords:** circummaxillary sutures, skeletal class iii, facemask, alt-ramec, rme

## Abstract

The correction of skeletal class III malocclusions is one of the most difficult orthodontic treatments. Skeletal Class III malocclusion may result from a combination of maxillary deficits and mandibular prognathism, mandibular prognathism alone, or maxillary deficits alone. Treatment options include an orthopedic appliance (facemask and chin cup), orthodontics with camouflage, a combination of orthognathic surgery and orthodontics, and the recently introduced bone-anchored maxillary protraction. This case report describes the treatment of a young, growing patient with a retrognathic maxilla using Hyrax with an acrylic splint using the alternative rapid maxillary expansion and constriction protocol and a Petite type facemask.

## Introduction

Skeletal or dental anomalies may lead to Class III malocclusion, which may impair the appearance and functionality of individuals. Significant variations have been observed in the occurrence of Class III malocclusion among different ethnic groups. Compared with the European population (4.88%), the prevalence of Class III malocclusion is higher in the Southeast Asian population (15.80%) [1].

Maxillary retrognathism was observed in 65-67% of all Class III malocclusions [2]. This unfavorable growth pattern usually necessitates early intervention. Early treatment with a reverse-pull headgear combined with a rapid palatal expansion appliance can successfully correct a retrognathic maxilla.

The maxilla articulates with nine bones of the craniofacial complex, namely, zygoma, inferior nasal concha, frontal, nasal, lacrimal, ethmoid, palatine, vomer, and, occasionally, sphenoid. Palatal expansion affects the intermaxillary and circummaxillary sutures, causing the maxilla to migrate forward and downward. Breaking these sutures may aid in the initiation of a cellular response to protraction forces [3].

A weekly regimen of alternating rapid maxillary expansion (RME) and constriction has been described in a previous study. Compared with traditional RME, this regimen permits a wider opening of the circummaxillary sutures [4].

## Case presentation

**Figure 1:**
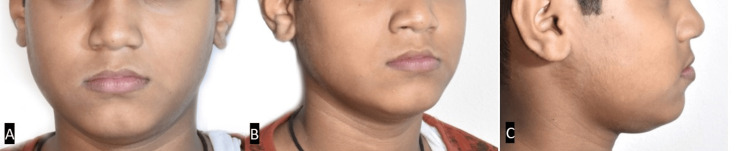
Pretreatment extraoral photographs. (A) Frontal view. (B) Oblique view. (C) Profile view.

A 12-year-old male patient came to the Department of Orthodontics with the chief complaint of irregularly placed upper front teeth. Extraoral examination revealed a straight profile and slightly obtuse nasolabial angle with a decreased Frankfort mandibular plane angle (Figure 1).

**Figure 2:**
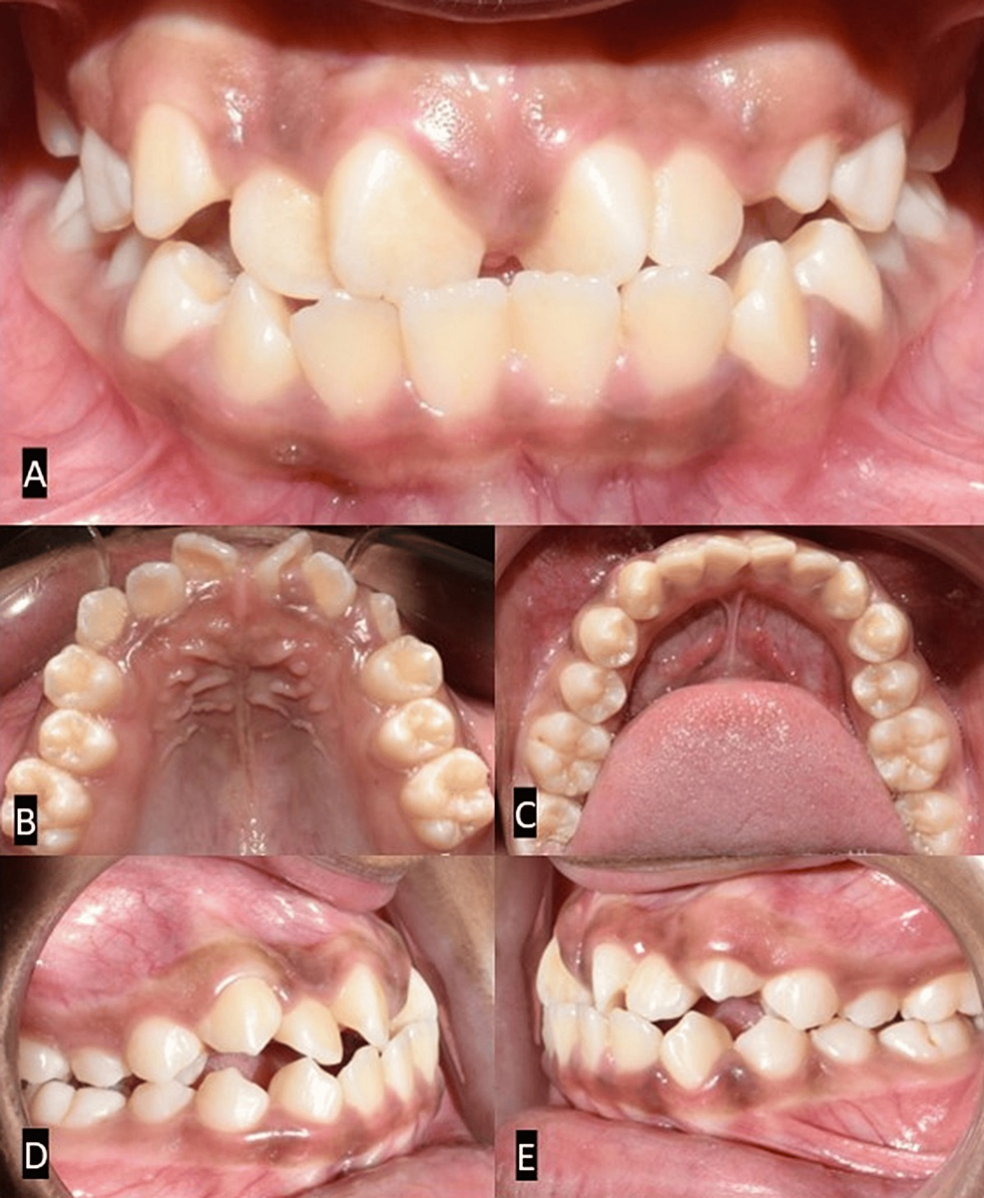
Pretreatment intraoral photographs. (A) Frontal view. (B) Occlusal view (maxilla). (C) Occlusal view (mandible). (D) Right occlusion. (E) Left occlusion.

Intraoral examination revealed an Angle Class III subdivision with a Class I molar relationship on the right side and a Class III molar relation on the left side. A midline diastema was noted along with rotation in 11, 12, 21, and 22. The patient had a reverse overjet of 2 mm, and a crossbite was noted in 15 and 25. Crowding was also observed in the lower arch (Figure 2).

**Figure 3:**
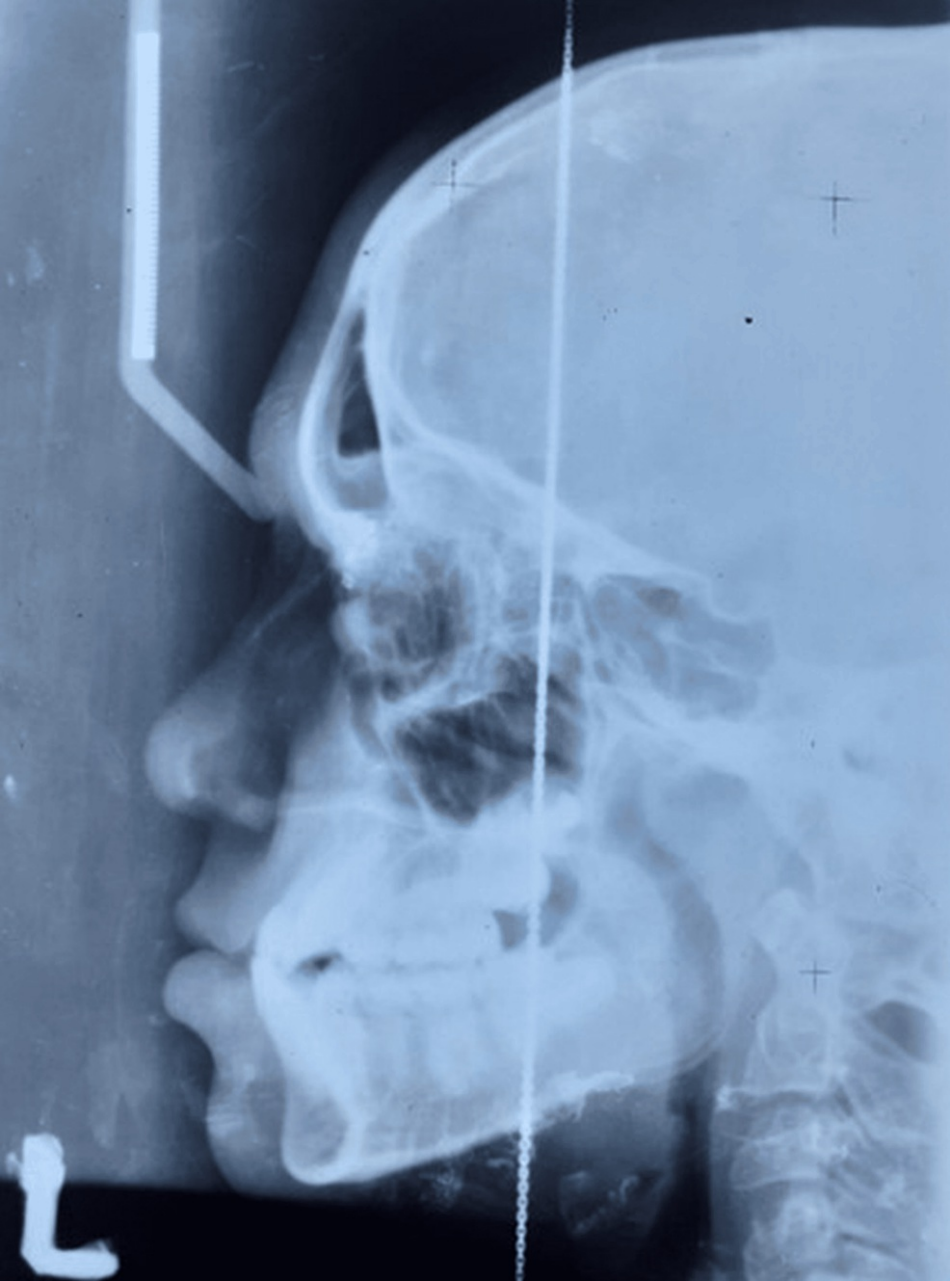
Pretreatment radiograph: lateral cephalogram.

The cephalometric findings of the patient indicated a skeletal Class III with retrognathic maxilla and horizontal growth pattern (Figure 3).

Treatment plan

**Figure 4:**
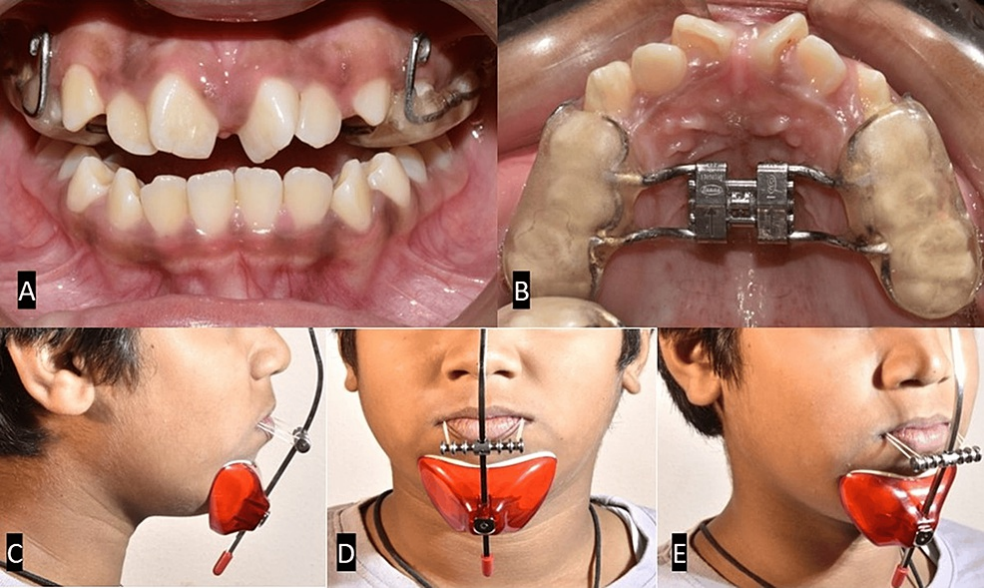
(A, B) Intraoral photographs at the end of alternative rapid maxillary expansion and constriction treatment. (C-E) Extraoral photographs taken at the time of placing the Petite facemask appliance.

The treatment plan was based on the patient’s chief complaint and correction of the underlying maxillary retrognathism. A Hyrax with an acrylic splint was placed on the maxillary posteriors to make way for the erupting canines and produce more bodily movement during expansion. Instead of the regular RME protocol, the Hyrax expansion appliance was activated based on the alternative RME and constriction (Alt-RAMEC) protocol (i.e., seven weeks of Alt-RAMEC of 1 mm/day). Following the alternate expansion and constriction, a Petite type facemask was used. Extraoral elastics were engaged bilaterally from the hook of an acrylic splint to the crossbow of the facemask (Figure 4).

**Figure 5:**
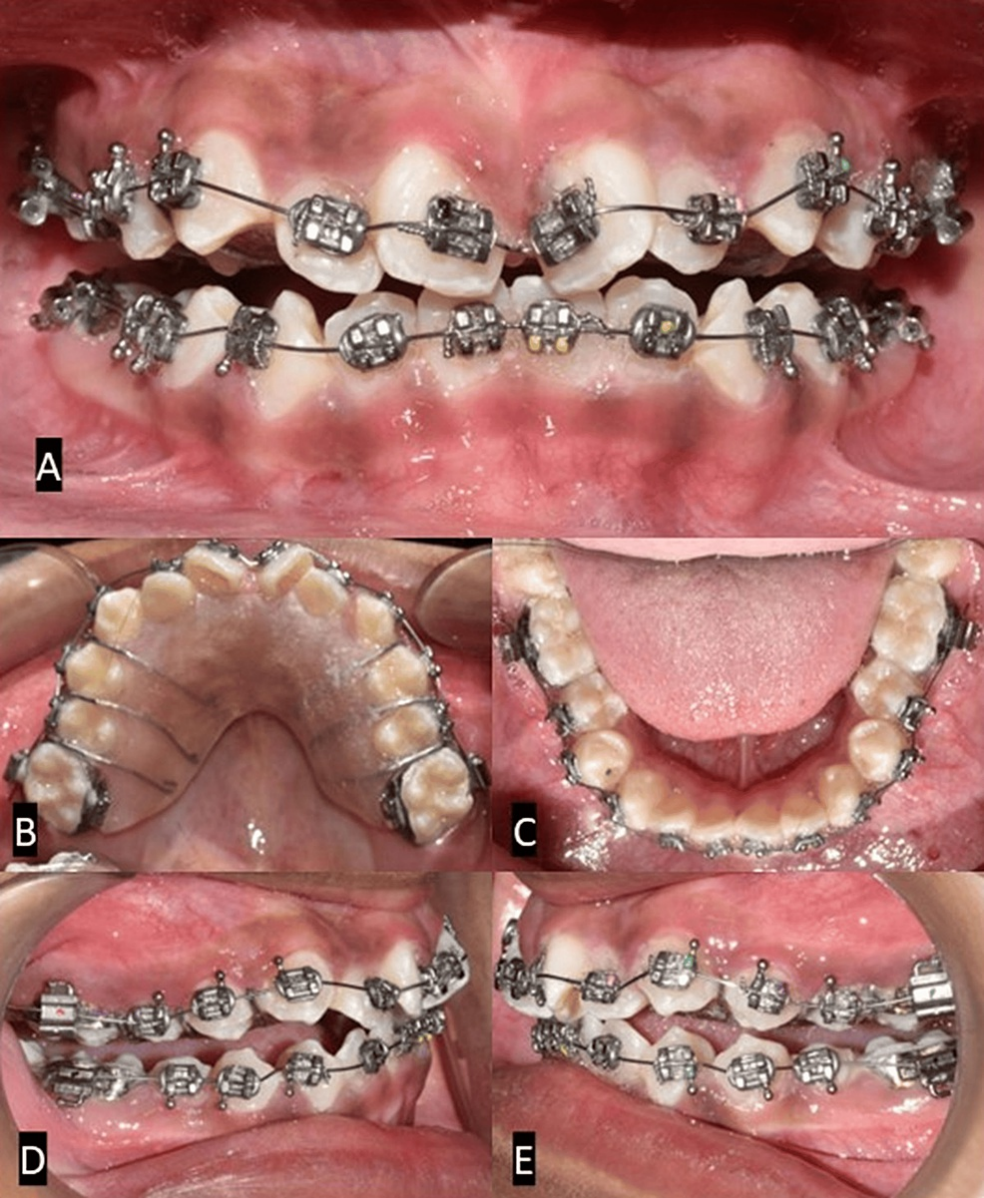
Intraoral photographs of leveling and aligning after Phase I (orthopedic phase) treatment. (A) Frontal view. (B) Occlusal view (maxilla). (C) Occlusal view (mandible). (D) Right occlusion. (E) Left occlusion.

After the correction of the anterior crossbite by achieving positive overjet, the upper and lower arch first molars were banded along with an acrylic plate to maintain the achieved transverse width. Bonding was done using a 0.022 slot McLaughlin, Bennett, and Trevisi appliance. The Initial leveling and alignment were performed using a 0.012 nickel-titanium (NiTi) wire, followed by 0.016 NiTi, 0.016 stainless steel (SS), 0.017 x 0.025 NiTi, 0.017 x 0.025 SS, and 0.019 x 0.025 SS (Figure 5).

**Figure 6:**
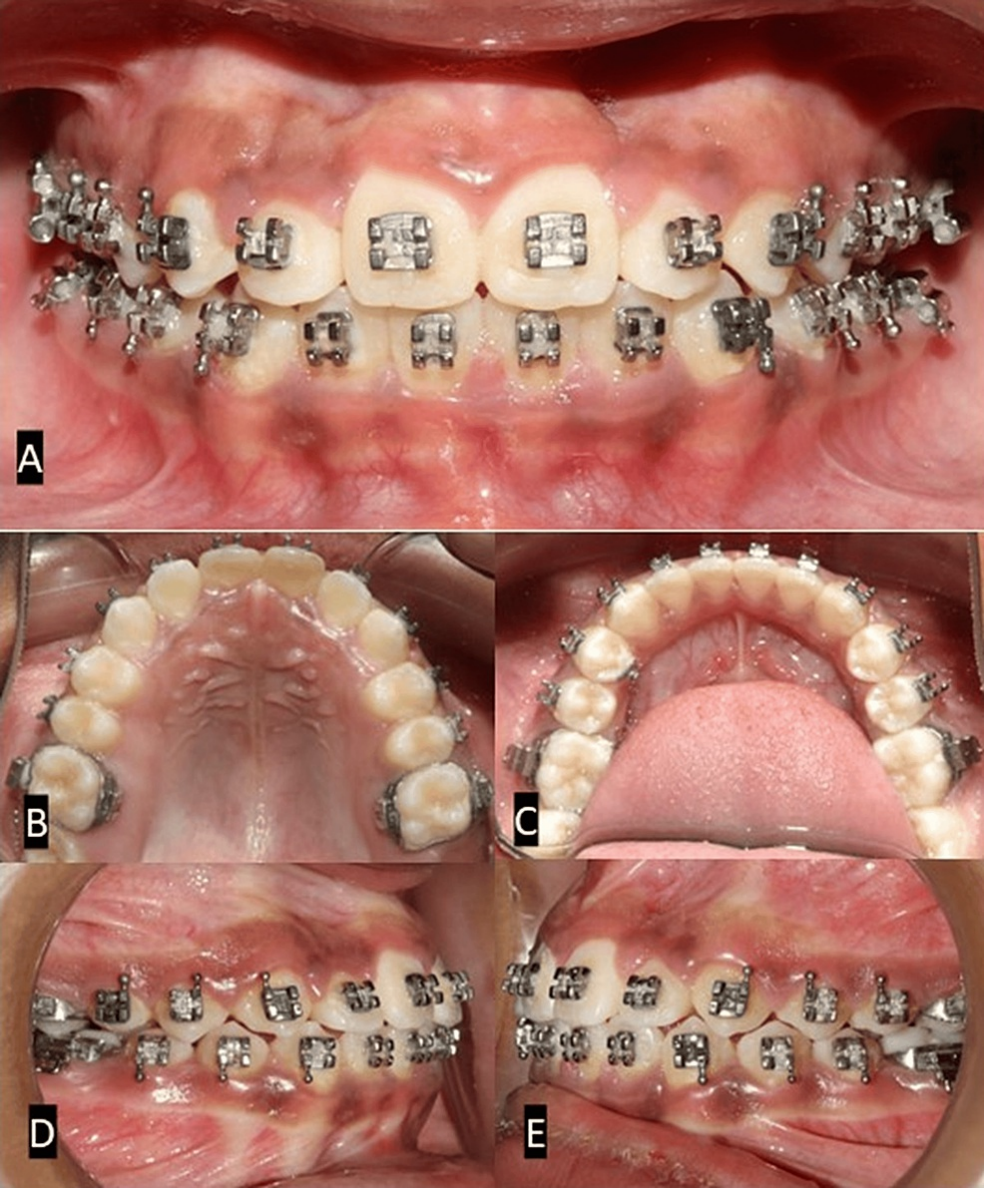
Intraoral post-treatment photographs. (A) Frontal view. (B) Occlusal view (maxilla). (C) Occlusal view (mandible). (D) Right occlusion. (E) Left occlusion.

**Figure 7:**
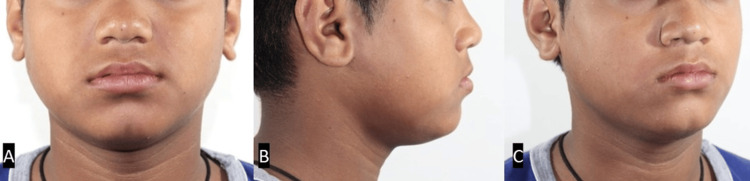
Extraoral post-treatment photographs. (A) Frontal view. (B) Profile view. (C) Oblique view.

Finishing and settling were performed using a 0.014 SS wire. Good intraoral intercuspation and axial inclination of the anteriors with normal overjet and overbite were achieved. The midlines coincided in the upper and lower arches, and the canines were Class I on both sides (Figure 6). The post-treatment results were satisfactory, with an ideal facial profile (Figure 7). The total treatment duration was 18 months. After the orthodontic treatment, the patient was advised to wear a chin cup appliance to restrict mandibular growth until its growth ceases.

## Discussion

Interceptive orthodontics aims to halt the progression of malocclusion and reroute it toward physiological development. Intercepting Class III malocclusion can prevent irreversible changes in the soft and hard tissues, such as gingival recession, overclosure of the mandible, labial wearing of the mandibular incisors [5], and overclosure of the anterior region of the labium [6,7], which usually requires orthognathic surgery in certain cases. By attaining aesthetics, a face mask or chin cup orthopedic therapies improve the skeletal component, reduce dentoalveolar compensation, enhance occlusal harmony, and help the patient psychologically [8].

When maxillary insufficiency was recognized as the primary factor contributing to skeletal Class III malocclusion, the use of facemask therapy in orthodontics increased. Maxillary forward movement and sutural remodeling are the mechanisms of action of facemask therapy. It is widely acknowledged that facemask therapy may be most effective at an early age. The growth potential of circummaxillary sutures at an early age can be used to treat maxillary deficiency instead of orthognathic surgery.

According to Liou and Tsai [4], compared to a single course of RME, repeated weekly treatment with Alt-RAMEC improved maxillary protraction three times and the anterior displacement of the maxilla twice. A study by Akbulut et al. [9] revealed that correction of the retrognathic maxilla was achieved within a short period when the Alt-RAMEC protocol was used.

According to Yagci and Uysal [10], there was more anterior advancement and bodily movement of the maxilla without much rotation when expansion was used along with facemask therapy. Kilic et al. [11] reported that patients with retrognathic maxilla who were treated with facemask therapy showed anterior advancement of the maxilla and a concomitant increase in upper lip thickness in children with growth potential. Moreover, there was a protrusion of the upper lip by a mean of 1.15 mm.

Yavuz et al. [12] assessed alterations in the maxillary sutures after facemask therapy in a 16-year-old female patient using single-photon emission computerized tomography. They found a significant increase in stimulated bone activity on both sides of the zygomaticomaxillary suture and outer zygomaticomaxillary area.

Yavuz et al. [13] reported that face mask therapy improved skeletal Class III malocclusion through a combination of skeletal and dental changes when used with or without RME. This indicates that RME should be used depending on the clinical scenario and is not mandatory to assist in the correction of maxillary retrognathism.

Tuba et al. [14] found that skeletal Class III treatment with facemasks with or without expansion produced similar skeletal changes. Minimal eruption of the upper molar was observed when an acrylic cap splint was used with an expansion appliance.

The upper and lower dentitions were well aligned with Class I canine and Class I molar relationships on both sides. The upper and lower midlines coincide perfectly with each other. Table 1 compares the pre- and post-treatment cephalometric readings. There was a mild increase in upper incisor inclination, which was negligible, whereas the lower incisors were ideally inclined at the end of the treatment. There was a mild variation in the nasolabial angle at the end, which was more favourable, and resulted in a better aesthetic profile.

**Table 1:** Pre-treatment and post-treatment cephalometric readings.

Cephalometric readings	Pre-treatment	Post-treatment
Sella nasion point A	79°	82°
Sella nasion point B	80°	80°
Point A nasion point B	-1°	2°
Sella nasion gonion gnathion	29°	31°
Frankfort-mandibular plane angle	22°	25°
Incisor mandibular plane angle	89°	90°
Upper 1 nasion point A angle	34°	35°
Upper 1 nasion point A linear	6 mm	6 mm
Lower 1 nasion point B angle	23°	25°
Lower 1 nasion point B linear	4 mm	4 mm

## Conclusions

A case of skeletal Class III with Angle Class III subdivision with reverse overjet and posterior crossbite in teeth 15 and 25 was successfully treated with a facemask and RME using the Alt-RAMEC protocol. Proper planning and diagnosis of malocclusion helped achieve positive treatment outcomes.
